# 

**Published:** 1885-06-20

**Authors:** 


☞ **Notice.**--- Subscribers in arrears are requested to remit their dues at once. Please take due notice and govern yourselves accordingly.

### EDITORIAL NOTICES.

CHOLERA is said still to linger at Marseilles, but the cases are not numerous.

MEDICAL IMPOSTER —A swindling doctor is reported to be traveling around telling a piteous story about a lost pocket-book, and thus procuring money from brother doctors. Look out for him.

DISFRANCHISING THE POOR.—A singular law has been adpoted in England, disfranchising all persons who have accepted medical relief from the parish. Such a law in the Southern States of the Union would in a short time abrogate negro suffrage in the cities of the South.

CANCER.—It is noticeable that the attention of the regular profession is being turned to the possibility of dealing successfully with malignant affections. We have always believed that a constitutional remedy would some day be discovered for cancerous affections, and we are glad to note that two hospitals for the special treatment of skin and cancer affections are being established in New York City.

A QUARREL.—A somewhat spicy correspondence recently occurred between Dr. Jacobi, of New York, a new code man, and Dr. J. V. Shoemaker, of Philadelphia. Shoemaker is reported to have said that the new code man had made threats at the Medical Congress, at Copenhagen, "that unless they were recognized they would use their influence to prevent the Congress from holding its next meeting in the United States." Several sharp notes passed, in which, according to Dr. Jacobi, Dr. Shoemaker "tries to wriggle out of his responsibility." A reply to this last was returned to Dr. Shoemaker, unopened.

### *RECEIPTED.*

1885.—B. M. Wooley, J. J. Stephens, A. T. Park, A. V. Ponder to June, J. M. Stansell, R. M. Savage to July, E. A. Speed, *Amos Kendall, Robt. E. Pharr, M. A. Mells, T. A. Wood, L. A. Boswell, J. Judkins.*

1884.—J. H. Sykes, J. P. Mell to March, C. F. Yeager, Jonathan Jones.

1882.—R. B. Stapleton to July.

1886.—L. M. Wood to July, *Amos Kendall to May, Seth Thomas to April, Miller Brooks to April,* John A. Meiggs to May.

